# Fabrication, Properties, Performances, and Separation Application of Polymeric Pervaporation Membranes: A Review

**DOI:** 10.3390/polym12071466

**Published:** 2020-06-30

**Authors:** Luchen Wang, Yan Wang, Lianying Wu, Gang Wei

**Affiliations:** 1 College of Applied Technology, Qingdao University, Qingdao 266100, China; wlch0313@163.com; 2 College of Chemistry and Chemical Engineering, Qingdao University, Qingdao 266071, China; yanwang@qdu.edu.cn; 3 College of Chemistry and Chemical Engineering, Ocean University of China, Qingdao 266100, China

**Keywords:** polymeric membrane, separation techniques, pervaporation, environmental science, hybrid materials

## Abstract

Membrane separation technologies have attracted great attentions in chemical engineering, food science, analytical science, and environmental science. Compared to traditional membrane separation techniques like reverse osmosis (RO), ultrafiltration (UF), electrodialysis (ED) and others, pervaporation (PV)-based membrane separation shows not only mutual advantages such as small floor area, simplicity, and flexibility, but also unique characteristics including low cost as well as high energy and separation efficiency. Recently, different polymer, ceramic and composite membranes have shown promising separation applications through the PV-based techniques. To show the importance of PV for membrane separation applications, we present recent advances in the fabrication, properties and performances of polymeric membranes for PV separation of various chemicals in petrochemical, desalination, medicine, food, environmental protection, and other industrial fields. To promote the easy understanding of readers, the preparation methods and the PV separation mechanisms of various polymer membranes are introduced and discussed in detail. This work will be helpful for developing novel functional polymer-based membranes and facile techniques to promote the applications of PV techniques in different fields.

## 1. Introduction

Membrane separation has emerged as one of the rapidly developing technologies in environmental science in the past few decades. Compared to traditional separation technologies, the membrane separation technique possesses superior performance, which has attracted tremendous attention in the separation community. It refers to the employment of a semi-permeable membrane to partially separate the feed containing two or more components, in which one or more components of the feed moves faster than the other components of the feed. It is well known that a membrane is a thin layer of natural or synthetic material holding selective separation function, which can separate target components of the solution.

Representative membrane separation technologies that are frequently adopted in various separation applications include reverse osmosis (RO), ultrafiltration (UF), electrodialysis (ED), nanofiltration (NF), membrane distillation (MD), and pervaporation (PV). Among these technologies, PV has been proved to be a rapidly developing technology for membrane-based separation [1]. PV is a process that has common elements with RO and membrane gas separation. In addition, PV also has many similarities with the steam permeation, which uses gas components on the feed side of a membrane. In contrast to steam permeation in which the flux of the steam strongly relies on the feed pressure, the PV flux is independent of the feed pressure [2]. Therefore, PV can be utilized to separate water from the organic liquid by partial evaporation with a porous membrane. The membrane in PV acts as a selective barrier between two phases, i.e., the liquid feed and the vapor permeation, which allows the designated components of the liquid feed to be transferred by evaporation [3]. The PV separation is nearly independent on the vapor-liquid equilibrium, since the permeation resistance lies on the adsorption equilibrium and the mobility of the permeation components in the membrane. The separation mechanism of PV primarily depends on the preferential adsorption and diffusion of target components through a membrane. The permeate side of the membrane is kept in a vacuum, while the feed side of the membrane is kept in atmospheric pressure or high pressure, thus creating a pressure difference on the membrane to maintain the driving force within the PV process. Therefore, PV is not restrained by the thermodynamic vapor–liquid equilibrium.

Compared with traditional separation technologies, PV not only has mutual advantages such as small floor area, simplicity, and flexibility, but also has unique characteristics including low cost, low energy consumption as well as high efficiency. It has been considered as an effective and energy-saving technology for separating those mixed chemicals that are difficult to achieve via conventional methods [4]. This technology has better separation capability and energy efficiency, which can lead to 40–60% energy saving [5]. In addition, PV does not require entrainers, thus it will not contaminate the original mixture [6].

To date, PV possesses broad application prospects and is marketed in petrochemical, desalination, medicine, food, biotechnology, and other industrial fields. It can be used to break azeotropes dehydration, organic/organic separation, acid separation and wastewater purification [7]. In the process of PV, membranes play a crucial role. In recent years, different types of membranes have been developed for PV, including polymers, ceramics, and composite membranes [8,9,10,11]. Among these membranes, polymer separation membrane exhibits distinct functions such as substance separation, recognition, energy conversion and substance transfer, which has been widely applied in various membrane separation processes. At present, the polymer separation membrane has been developing towards the direction of high efficiency and selectivity, functional compounding and diversification. A large number of studies are now focused on exploring polymer PV membranes with high selectivity, high flux, flawless and large-scale preparation.

In this paper, we review the recent development in the fabrication of polymeric PV membranes for separation technology. In Section 2, the synthesis and properties of polymeric PV membranes are introduced as synthesis methods, types of polymeric membrane and membrane module. In Section 3, characteristics of the PV membrane separation process are discussed in terms of membrane performance and energy utilization of PV. In Section 4, the PV membrane separation technology is introduced from the principle, mass transfer mechanism, operation mode, and influence conditions, as well as process simulation and optimization. Then, Section 5 summarizes the PV applications of polymeric membranes in the chemical separation, desalination, petrochemical, pharmaceutical, food, and biotechnology industries. Finally, the perspectives on the further development of polymeric membranes and PV processes are predicted.

## 2. Synthesis, Types and Properties of Polymeric PV Membranes

Polymeric membrane plays an important role in the PV process, thus in this section, we mainly introduce synthesis methods, functionalization, membrane module, membrane performance measurement standards and energy utilization.

### 2.1. Synthesis Methods

Various methods are available for the preparation of the PV membrane. In this sub-section, we summarized some of the representative techniques for creating polymeric membranes, including solution casting, hollow fiber spinning, solution coating, interface polymerization and membrane modification.

#### 2.1.1. Solid Solution Casting

**Figure 1:**
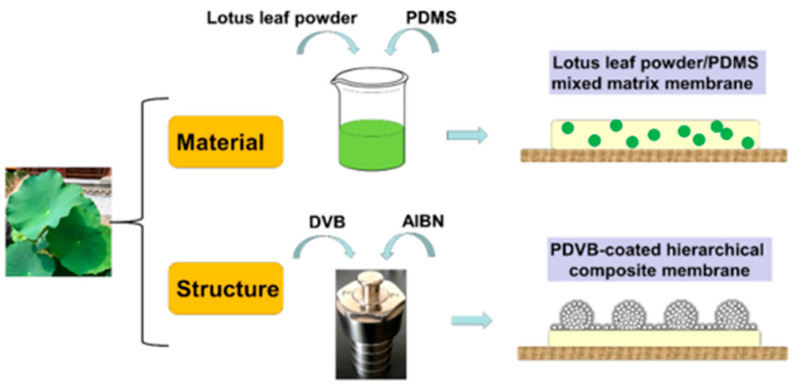
Preparation process of two kinds of lotus polydimethylsiloxane (PDMS) composite membranes. Reproduced with permission from Reference [12]. Copyright 2020, Elsevier.

Solution casting is the most commonly used method for the synthesis of the flat membrane. For example, He et al. [12] prepared two kinds of lotus polydimethylsiloxane (PDMS) composite membranes by solution casting method, namely lotus leaf powder/PDMS mixed matrix membranes (MMMs) and polydivinylbenzene coated PDMS composite membranes. The preparation process of the above PDMS composite membranes is illustrated in Figure 1. As PDVB also has preferential adsorption for ethanol, the PDVB-coated PDMS membrane showed a higher ethanol recovery performance with the separation factor and total flux increased by 13% and 30%, respectively. Therefore, it was demonstrated that PDVB coating is an effective method to fabricate superhydrophobic membranes and both the two lotus-inspired strategies are feasible in optimizing pervaporation performance for ethanol recovery. In addition, Shahverdi et al. [13] prepared poly(vinyl alcohol) (PVA)/zeolite 4A mixed matrix composite membranes supported on polypropylene microfiltration membranes by solution casting method and crosslinked with glutaraldehyde to investigate their PV separation properties of water–ethylene glycol mixtures. In another study, Flynn et al. [14] incorporated spherical, discreet, size-monodisperse mesoporous silica particles of 1.8–2 μm diameter, with pore diameters of ~1.8 nm into a PVA polymer to produce composite PV membranes. The selective membrane layers were cast on polyacrylonitrile (PAN)/non-woven fabric supports. 

#### 2.1.2. Hollow Fiber Spinning

Compared with the flat membrane, the hollow fiber membrane has the advantages of high filling density, self-supporting structure, and a self-contained vacuum channel. In the spinning process, when the primary fiber contacts with coagulant, the film is formed by phase transformation. As the polymer coating and the liquid in the inner hole of the primary fiber are extruded at the same time, the primary fiber solidifies on its inner surface immediately. However, due to the air humidity, when the primary fiber passes through the air gap area, part of it solidifies on the outer surface. With the development of the spinning method from single-layer to double-layer co-extrusion, the complexity of hollow fiber spinning is increasing, which is cost-effective for the membrane preparation, and it is more optional in the selection of materials and forms of the support layer [15,16]. 

**Figure 2:**
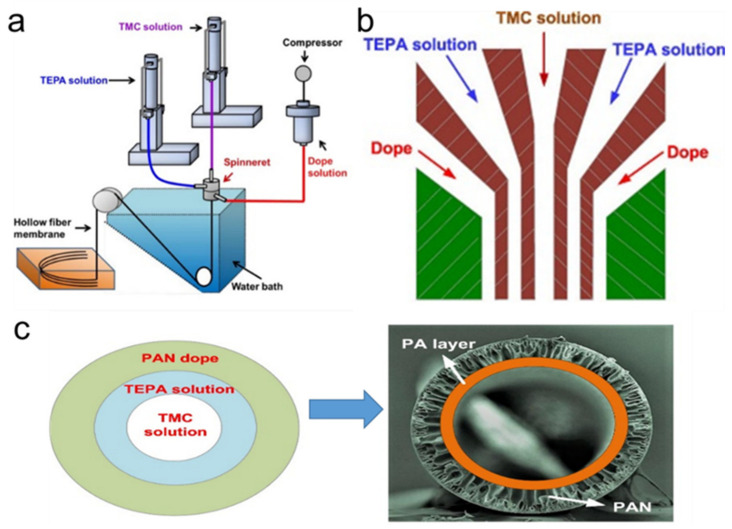
Schematic diagram of (**a**) PA/PAN composite hollow fiber membrane fabrication frame, (**b**) profile of the triple orifice spinneret, and (**c**) PA layer formation. Reproduced with permission from Reference [17]. Copyright 2018, Elsevier. PA, polyamide; PAN, polyacrylonitrile.

For example, Tsai et al. [17] explored a new method for the fabrication of polyamide (PA)/ PAN composite hollow fiber membrane by using a triple orifice spinneret. Tetraethylenepentamine (TEPA) and trimesoylchloride (TMC) were used as the monomers of aqueous solution and acid chloride solution, respectively. The PAN dope, TEPA solution, and TMC solution were pumped into the outermost, middle, and inner channel of the triple orifice spinneret respectively, and then co-extruded into the water from the outlet of the spinneret simultaneously. Then, the PA layer was formed on the lumen surface of the synchronous wet-spun PAN hollow fiber membrane. The schematic diagram of PA/PAN composite hollow fiber membrane fabrication apparatus and PA layer formation are revealed in Figure 2.

#### 2.1.3. Solution Coating

The solution coating is often used for depositing a thin selective layer on the microporous substrate or support to prepare the composite membrane. Among these substrates or supports can be flat, hollow fiber or tubular structure, but the substrate must be completely porous to minimize the structural resistance, so that the membrane resistance is mainly controlled by the coating selective layer [18,19]. The pore size distribution of the substrate surface shall be tight and free from large defects to prevent the invasion of the coating solution. Before coating, the substrate is pre-wetted with a low boiling point solvent incompatible with the coating solvent, which minimizes the risk of intrusion. Therefore, the pre-wetting solvent is removed by drying to obtain the coating film. It is a big challenge to coat the hollow fiber evenly, because the small-diameter hollow fiber with uneven coating will have a negative effect on the separation process.

**Figure 3:**
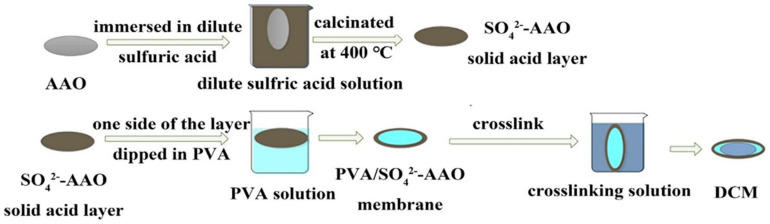
Schematic diagram of pervaporation (PV) of DCMs. Picture of various DCMs Reproduced with permission from Reference [20]. Copyright 2020, Elsevier.

For example, Sun et al. [20] prepared a polyvinyl alcohol/SO_4_^2−^-anodic aluminum oxide (AAO) membranes. Firstly, the PVA/AAO pervaporation composite membranes (SCMs) were prepared by using PVA as the active separation layer and AAO as the support layer. Secondly, the SO_4_^2−^ was loaded in the pores of AAO by dipping the membranes in dilute sulfuric acid solution. Thirdly, the membranes were calcinated at 400 °C to produce the solid acid catalyst layer (SO_4_^2−^–AAO). Finally, PVA solution was coated on one side of SO_4_^2—^AAO. The PV process diagram of dual-functional flat composite membranes (DCMs) is illustrated in Figure 3.

#### 2.1.4. Interfacial Polymerization

The interfacial polymerization (IP) is more versatile than the other fabrication techniques, e.g., dip coating, photografting, and layer-by-layer self-assembly. Because high molecular weight polymers can be obtained even at mild reaction conditions, and polymerization proceeds rapidly [21]. IP is commonly applied in the preparation of RO, NF and PV composite films. 

**Figure 4:**
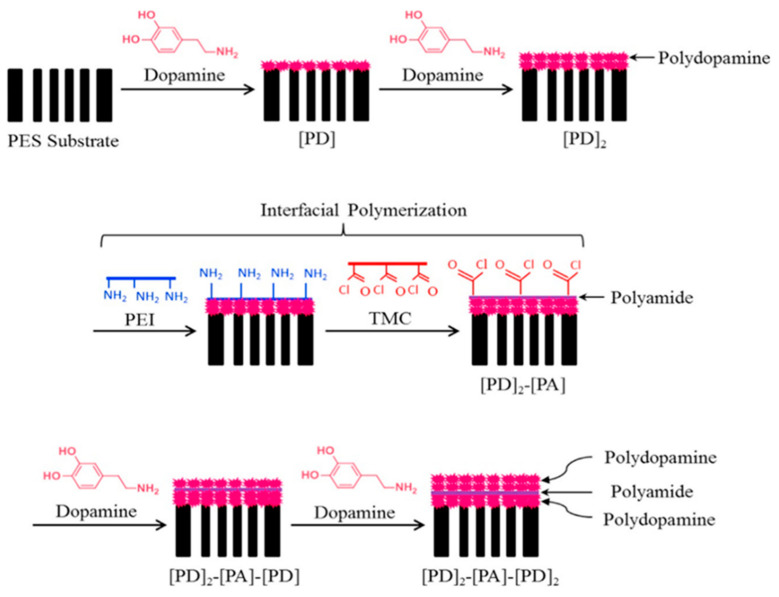
Schematic diagram showing the procedure to prepare thin film composite membrane two plies of polydopamine (PD), one ply of PA and two plies of PD formed on the substrate sequentially ([PD]_2_–[PA]–[PD]_2_) by PD deposition and interfacial polymerization (IP). Reproduced with permission from Reference [22]. Copyright 2015, Elsevier.

For example, Wu and co-workers [22] developed a novel thin-film composite membrane manufactured with sequential deposition of polydopamine (PD) via self-polymerization, and PA through IP for dehydration of ethylene glycol. They found that the PD layer acting as a transitional layer could increase PV membrane selectivity by enhancing the adhesion between the PA surface layer and the substrate. Figure 4 illustrates the process of membrane synthesis with sequential deposition steps for PD and PA formation by self-polymerization and IP, respectively. The composite membranes consisted of PA and PD sublayers, which was formed by IP from polyethyleneimine (PEI) and TMC at a PEI concentration of 4.0 wt % and a TMC concentration of 0.8 wt %. In another study, Cui et al. [23] fabricated a series of acid-resistant polysulfone/polyethersulfone composite membranes to purify acid wastewater, in which the IP was adopted for the preparation of self-supporting polysulfonamide films. The aqueous phase was a mixture of m-phenylenediamine and triethylenetetramine, and the organic phase was 1,3-benzene sulfonyl chloride n-hexane. Finally, the formation of a polymeric membrane can be found at the interface between n-hexane and water.

#### 2.1.5. Membrane Modification

**Figure 5:**
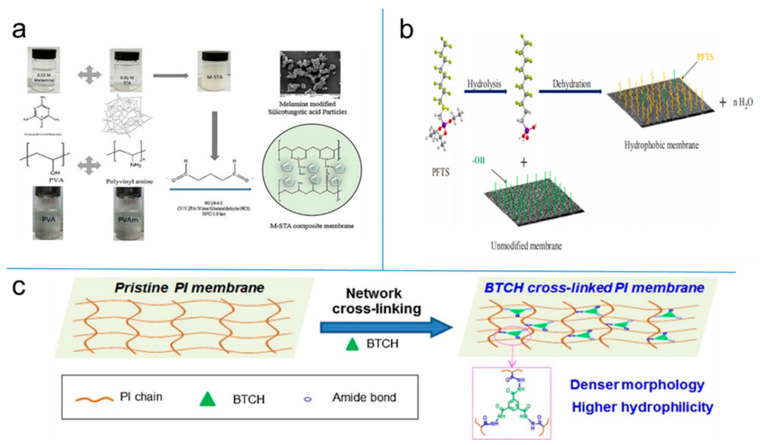
(**a**) Schematic diagram of melamine modified STA and PVA /PVAm blend film. Reproduced with permission from Reference [26]. Copyright 2018, Elsevier. (**b**) Schematic diagram of PFTs grafted powder/ film. Reproduced with permission from Reference [27]. Copyright 2020, Elsevier. (**c**) Schematic diagram of PI membrane crosslinking BTCH. Reproduced with permission from Reference [28]. Copyright 2017, Elsevier.

Structural and functional modifications of PV membranes are usually required to improve the performance and/or the stability of polymeric PV membranes. The chemical crosslinking is the most commonly used technology to stabilize the membrane and inhibit the swelling phenomenon. To inhibit the swelling problem, polyvinylalcohol (PVA) has been modified using different methods such as chemical crosslinking, polymer grafting, blending with different polymers, the formation of PVA copolymers, and thermal treatment [24,25]. The hydrophilicity and hydrophobicity of the polymeric membranes can be improved by adding or grafting related functional groups into the polymer chain. For example, silicotungstic acid (STA) was modified with melamine [26], and the MMMs containing melamine-modified STA (M-STA) particles were prepared for the PV dehydration of a water isopropanol (IPA) mixture. In the membrane durability study, the M-STA composite membrane displayed good stability and constant PV results for longtime PV operation, which confirmed that melamine modification prevented the leaching of STA from the membrane. Both poly(vinylamine) (PVAm) and PVA are hydrophilic, containing amine -NH_2_ and -OH functional groups in their main chains, consequently, the combination of these polymers may yield a high-quality PV membrane for use in the dehydration process. The preparation principle of melamine modified STA and PVA /PVAm blend film is shown in Figure 5a.

In a hydrophobic PV process, the polymeric membranes that grafted through chemical modification with 1H,1H,2H,2H-perfluorooctyltriethoxysilane (PFTS) were utilized. Asymmetric alumina supported zirconia grafted 1H,1H,2H,2H-perfluorodecyltriethoxysilane membranes were successfully prepared by Li et al. [27]. The effects of PFTS concentration, different solvents and alkali pretreatment on the hydrophobicity of the membrane surface were investigated, as shown in Figure 5b.

Besides, the amine cross-linking is one of the popular modifications of polyimide (PI) membranes in order to improve their operation stability and separation performance. Xu et al. [28] synthesized a novel kind of cross-linker tricarbohydrazide-1,3,5-benzene tricarboxylic acid trihydrazide (BTCH) and it was incorporated into PI membranes for IPA dehydration via PV, as indicated in Figure 5c. 

### 2.2. Types of PV Membranes

There are many types of PV membranes, which can be divided into homogeneous membrane, asymmetric membrane and composite membrane. Both the homogeneous membrane and the asymmetric membrane are made of the same material, however, the physical structure in the cross-sectional direction of the two membranes is different. The composite membrane is made of different materials, and in the cross-sectional direction of the membrane, the physical and chemical properties also are different. The homogeneous membrane is usually thick, with high resistance of the components and low permeability flux through the membrane, thereby it is often used in laboratory research. The asymmetric membrane is composed of a relatively thin porous dense cortex and a supporting layer, which has usually large flux and excellent separation effect. The composite membrane is similar to the asymmetric membrane in structure, while the main difference is that the composite membrane is made by covering a dense separation layer on the porous supporting layer [29]. 

Based on different membrane materials, organic polymer membrane, inorganic membrane and organic/inorganic composite membrane are often adopted as PV membranes. The organic membrane is usually cost-effective, but it is not resistant to high temperature, high pressure and poor stability in organic solvent. On the contrary, inorganic membranes exhibit good thermal stability, mechanical stability and solvent resistance. Nevertheless, the preparation costs of inorganic membranes are relatively high, which restrict their large-scale production. Hybrid materials usually combine organic and inorganic phases at the molecular level through chemical bonds [30], which have developed rapidly in recent years [31]. Owing to the combination of different materials, hybrid membranes possess distinct characteristics such as high stability, membrane structure modifiability and permeability. 

**Figure 6:**
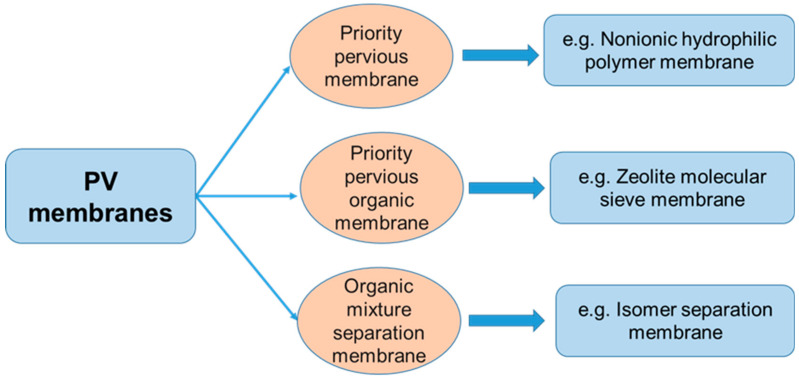
Classification of PV membranes.

PV membranes such as priority pervious membrane, priority pervious organic membrane and organic mixture separation membrane usually present different functions in the PV separation process (Figure 6), which have been extensively studied in recent years. For example, the separation cortex in the priority pervious membrane contains an adsorption center that can produce hydrogen bonding, ion coupling or coupling pole coupling with water molecules, hence this kind of membrane exhibits certain hydrophilicity. Polymer materials that are commonly used for the preparation of priority permeable membrane include cellulose acetate (CA) [32], chitosan, PI [33], polyacrylic acid and so on. Due to great differences in the properties of various organic mixture systems, the membrane materials for the separation membrane of organic mixtures are not as regular as the two processes, i.e., dehydration of organic substances and removal of organic substances from water. It is necessary to select and design membrane materials according to the differences in molecular size, shape and chemical structure of the mixture components. At present, materials of the PV membrane are usually selected empirically, as there is no mature standard for the selection of membrane materials. Polymer with high selectivity is usually the first choice for further research since the disadvantage of low permeability can be partially compensated by introducing asymmetry into the membrane structure, so as to reduce the effective thickness of the membrane.

### 2.3. Membrane Module

**Figure 7:**
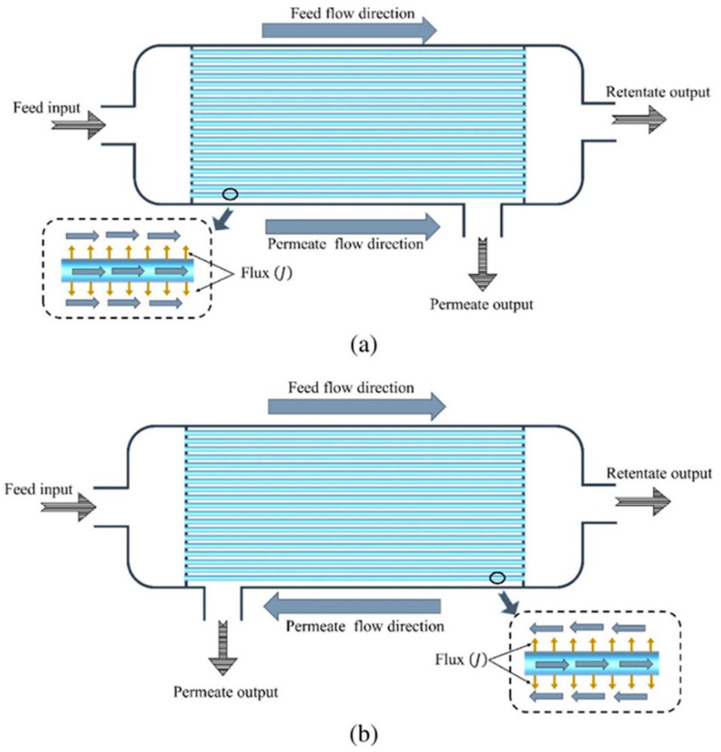
Flow pattern of (**a**) parallel flow and (**b**) counterflow of the hollow fiber membrane module. Reproduced with permission from Reference [34]. Copyright 2017, Elsevier.

Flat membranes, frame membranes and spiral wound membranes have been widely used in PV. Nowadays, the hollow fiber membrane module as a substitute of flat membrane has appeared in PV membrane separation technology, but it is still under development. The structure of the hollow fiber membrane module is similar to the shell and tube heat exchanger, which contains a large number of membrane fibers. The membrane fibers are encapsulated in the module shell and it has the advantages of high filling density and self-support. Both the parallel and the reverse flow modes of hollow fiber membrane module are presented in Figure 7. 

For example, removal of thiophenes from fluidized catalytic cracker gasoline by using a hollow fiber pervaporation module, the counter-current hollow fiber modules have higher average total flux as well as higher average thiophene flux in comparison to co-current hollow fiber modules. Thus, the values of stage cut are slightly higher for counter-current hollow fiber module than co-current hollow fiber modules. However, the enrichment factor of thiophene is lower for a counter-current hollow fiber module than for a co-current hollow fiber module. Therefore, co-current hollow fiber modules are slightly more selective for thiophene than the same size of counter-current hollow fiber modules. However, the counter-current module is a better option than the co-current hollow fiber module in order to achieve maximum output from a single module [34]. At the feed side of the membrane, the liquid in contact often adsorbs in the membrane, leading to membrane expansion. This phenomenon can change the properties of the membrane, leading to higher permeability and lower selectivity. Due to the permeation side of the membrane, the vapor pressure of the components has a great influence on the permeation rate, so the downstream steam pressure must be kept at the lowest level under the premise of economic feasibility to maximize the driving force of infiltration. These phenomena need to be considered in the design of the hollow fiber membrane unit. 

## 3. Characteristics of the PV Membrane Separation Process

### 3.1. Performance of PV Membrane Separation Process

In the process of PV membrane separation, PV flux, separation factor, separation index and separation efficiency are usually used to evaluate the separation performance.

(1) Permeate flux

Membrane productivity is the number of components penetrating through a specific area of the membrane surface in a given time unit. The production capacity of the membrane is usually measured by the permeation flux J, which relates the product rate to the membrane area required to achieve separation [35]. Equation (1) is the expression of the J.

(1) $$J=\frac{V}{\mathrm{St}}$$

where, V is the total amount of the permeate (L or kg), S is the effective surface area of membrane (m^2^), and t is the permeation time (h).

(2) Separation factor

When describing a mixture consisting of component A and other components, the separation factor α of component A is defined as:

(2) $$\alpha =\frac{y_{A}\left(1-x_{A}\right)}{x_{A}\left(1-y_{A}\right)}$$

where x_A_ and y_A_ are the mole fractions of component A in the feed and permeate respectively. And when α=1, the membrane has no separation ability for components A and B. α>1, component A is easier to penetrate than B. α→∞, components are completely separated.

(3) Separation index

The product of J and α is called the separation index (PSI), which can be used to evaluate the overall performance of the membrane. The expression PSI can be expressed as follows:

(3) $$\mathrm{PSI}=\left(\alpha -1\right)J$$

(4) Thermal efficiency

Thermal efficiency can be defined as the ratio of the effective output energy to the input energy. Q*_C_* is the heat that permeates through the components of the membrane, and its size is directly proportional to the flux. Q*_L_* is the heat conduction quantity caused by both sides of the membrane, and its size is directly proportional to the temperature difference, but both are inversely proportional to the thickness of the membrane. In the PV process, the heat conduction direction of Q*_L_* is opposite to the mass transfer direction, hence the heat efficiency of PV (η_pv_) is given as:

(4) $$\eta_{\mathrm{pv}}=\frac{Q_{C}}{Q_{C}-Q_{L}}$$

### 3.2. Energy Utilization of PV Membrane Separation Process

Energy consumption is considered as a significant factor for the desalination process, as the supply of saltwater and energy are directly related to the production of drinking water. Representative desalination technology is RO, which uses a semipermeable membrane with a pressure typically ranging from 5.5 to 6.8 MPa and a minimum energy consumption of around 2 kWh/m^3^ [36]. Other feasible desalination methods including MD [37], multi-stage flash [38] and PV [39] have lots of advantages and disadvantages respectively. In this section, three desalination technologies, i.e., RO, MD and PV are compared in terms of the energy consumption, as presented in Table 1.

MD is usually carried out at a temperature of 50–90 °C and it can utilize conventional low-temperature sources such as solar, geothermal and residual heat [39]. However, PV involves phase transformation. When low-grade heat sources (i.e., solar energy, geothermal energy or industrial waste heat) can effectively heat the feed to the range of 40–75 °C, PV desalination will be more competitive [32], and the energy requirements of a PV plant also are much lower [40].

**Table 1:** Experimental water flux and energy consumption of the desalination process with RO, membrane distillation (MD) and PV.

Process	Water Flux [kg] (m^−2^ h^−1^)	Reference	Energy Consumption (kWh m^−3^)	Reference
RO	37.0	[41]	3.5	[42]
	60.0	[43]	2.0	[44]
PV	15.0	[45]	2.0	[46]
MD	6.5	[47]	1.1	[47]
	10.5	[48]	1.25	[49]

Table 1 data is considered only to the electrical energy required for pumping the feed and the permeate and does not include the energy needed to warm up the feed. However, the exemplary values of energy consumption given in Table 1 prove that the results obtained from experimental data differ significantly and as such are not reliable enough to assess the industrial energy consumption.

Among these three desalination methods shown in Table 1, RO has high energy consumption because it requires high operating pressure. In the actual industrial production, MD and PV require heating the feed liquid, so they need to consume more energy. The water flux of MD is much smaller than RO, and its low energy consumption may be caused by the low temperature of feed liquid. However, both water flux and energy consumption of PV is between RO and MD. The difference between PV and RO is that energy must be added continuously during the operation process because there are phase transitions and negative pressure downstream of the PV membrane. The energy efficiency of desalination is determined by the availability of renewable energy sources necessary to raise the feed temperature. Traditional desalination system consumes a relatively large amount of energy [50,51]. When obtaining energy from fossil fuels, desalination inevitably involve greenhouse gas emissions, which should be considered [52,53].

Another alternative method that has shown high potential for energy efficient desalination is PV. The high energy conservation efficiency of the PV process along with its ability to handle high salinity, high salt rejection and lower cost make the process suitable for desalination [54]. The energy efficiency is primarily determined by the availability of the renewable energy sources, such as solar or waste heat, etc., that is required to heat the feed solution. The electrical energy utilization for the pumping of the feed and the permeate water also contribute a significant amount of energy consumption during the desalination process [36]. Moreover, the potential of RO system for desalination is only limited up to 50%. In fact, pervaporative desalination of the seawater is considered to be the potential alternative methods for solving the water scarcity owing to several advantages, such as high energy conservation at the expense of low cost, high salt rejection efficiency (~100% of salt rejection) [55] and better handling ability of water with high salinity.

## 4. Mechanism, Operation Mode and Numerical Simulation of the PV Membrane Separation Process 

This section summarizes the PV membrane separation technology including principle, mass transfer mechanism, operation mode, influence conditions, process simulation and optimization.

### 4.1. Principle

**Figure 8:**
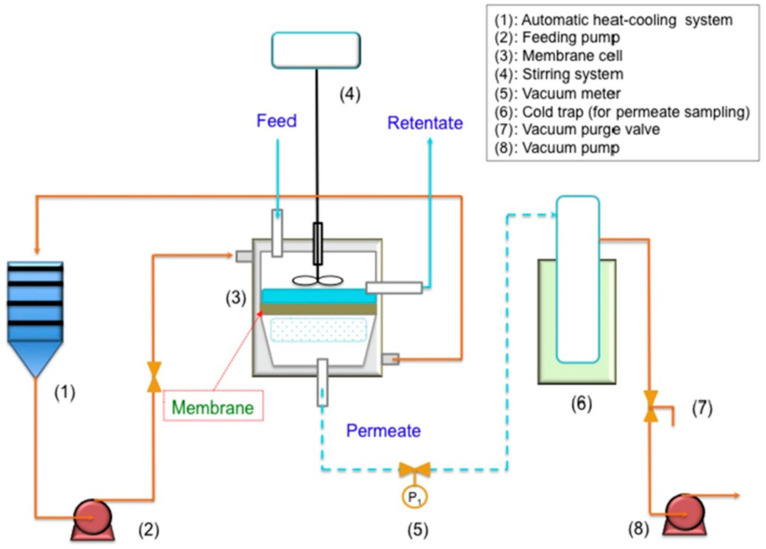
Equipment flow chart of the PV process. Reproduced with permission from Reference [56]. Copyright 2019, Elsevier.

After the feed liquid enters the PV membrane module, the membrane is divided into the feed side and the permeation side. The feed side is under atmospheric pressure while the permeate side maintains a lower partial pressure by vacuuming or carrier gas purging. Due to the chemical potential gradient (vapor differential pressure) of each component on both sides of the membrane, the components in the feed liquid pass through the membrane and vaporize on the permeation side. Because of the different physical and chemical properties between the components, their movement rate in the PV membrane is different. The components that are easy to permeate can be separated from the raw material liquid preferentially and concentrated on the permeate side. While the components that are not easy to permeate are accumulated in the raw material liquid to form the separated retentate, so as to realize the separation of the raw material liquid. The flow chart of PV equipment is presented in Figure 8.

### 4.2. Mass Transport Mechanism

**Figure 9:**
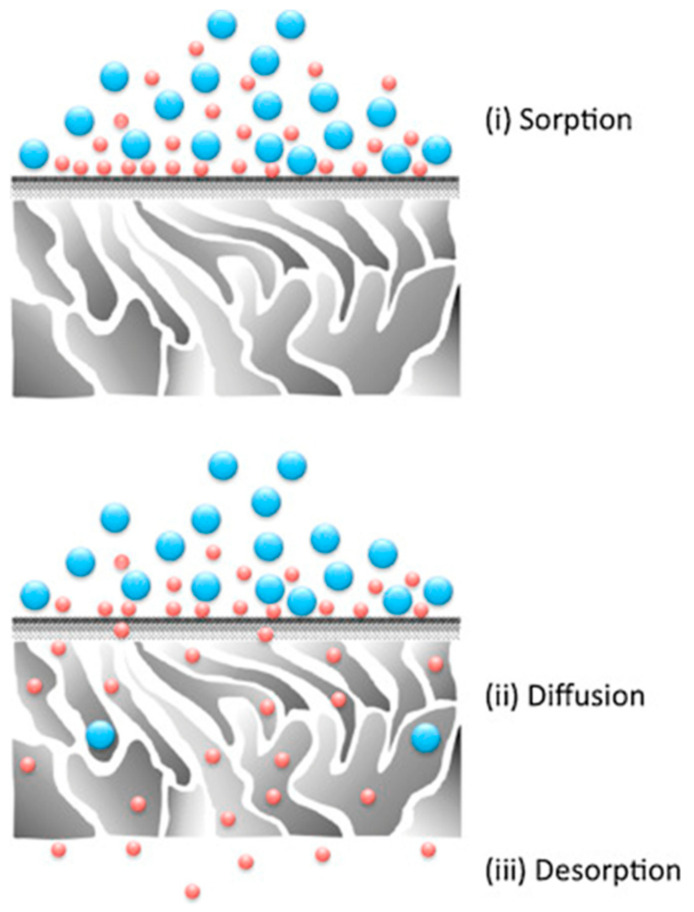
Schematic diagram of the dissolution diffusion model process. Reproduced with permission from Reference [62]. Copyright 2016, Elsevier.

PV is a complex process that includes mass transfer and heat transfer at the same time. The models used to describe the mechanism of the transfer process include the solution-diffusion model [57], pore flow model [58], virtual phase transition solution-diffusion model [59], and irreversible thermodynamics model [60]. Among the most widely accepted is the solution-diffusion model. PV dissolution diffusion process can be divided into the following three steps [35,61]: (I) the dissolution of each component in the solution on the membrane surface at the upstream side of the membrane; (II) the diffusion from the upstream to the downstream of the membrane in the form of molecular diffusion under the action of chemical potential difference; (III) the PV of components on the downstream side of the membrane at low vapor pressure. The process diagram of the dissolution diffusion model is illustrated in Figure 9.

### 4.3. Operation Mode of PV Process

**Figure 10:**
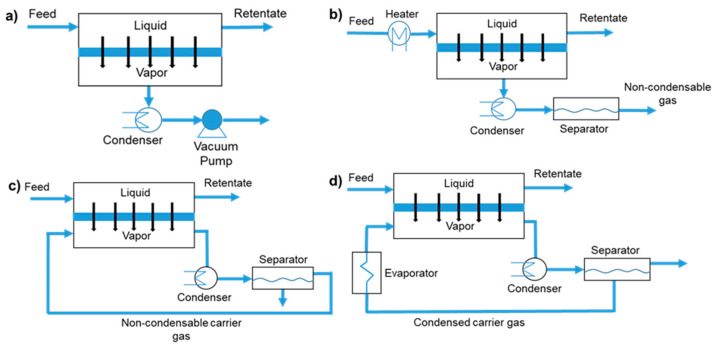
Operation mode of PV process: (**a**) vacuum PV; (**b**) thermal PV; (**c**) carrier gas purging PV; (**d**) condensable carrier gas purging PV.

To maintain the vapor pressure difference between upstream and downstream components of the membrane, the PV process mainly includes vacuum PV, thermal PV, carrier gas purging PV, and condensable carrier gas purging PV. Different operation modes with their distinct characteristics and range of application are summarized in Table 2 and the different operation processes are shown in Figure 10.

### 4.4. Influence of Process Conditions

The PV process is mainly affected by intrinsic properties of the PV membrane such as material, structure and thickness of the membrane, and external conditions including temperature, pressure, concentration polarization, and sweep velocity. Several key factors that would affect the PV process are discussed as follows:

(1) Membrane properties. It was showed that the material of the membrane has significant impacts on the PV process. For example, if a small amount of water is to be removed from the organic matter, PVA/PAN composite membrane with better hydrophilic performance is often employed, as it is conducive to the dissolution and diffusion of water in the membrane. On the contrary, if a small amount of organic matter is to be removed from the water, hydrophobic composite membranes such as organosilicon can be selected [72]. Because the hydrophobic membrane is conducive to the dissolution and diffusion of a small amount of organic matter in the membrane, therefore, the energy consumption cannot be reduced until the dissolution and diffusion of water in the membrane are minimized. Besides, the thickness of the membrane also impacts the PV process, if the membrane is thicker, the resistance and flux of the permeation process will be larger and smaller respectively.

(2) External conditions. The operating temperature has a major influence on the PV process. With the increase of temperature, the expansion and free volume of the membrane, the diffusion coefficient of the permeate molecules, the driving force on both sides of the membrane, and the permeation flux of the membrane are increased, but the viscosity of the permeate is decreased, [52,73,74,75]. Besides, upstream and downstream pressure changes can affect the flux of the PV process as well.

**Figure 11:**
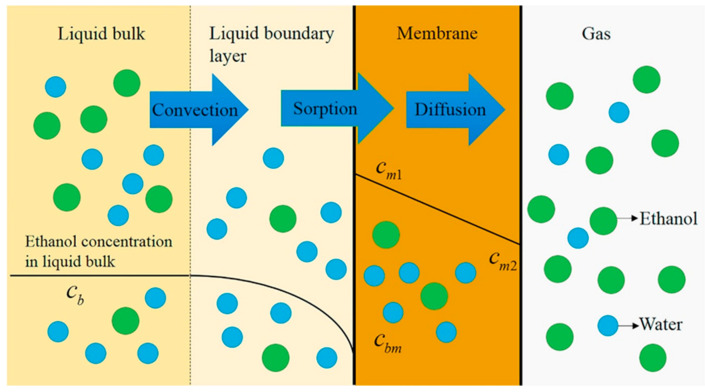
Scheme of ethanol mass transfer considering concentration polarization during PV. Reproduced with permission from Reference [76]. Copyright 2019, Elsevier.

Concentration polarization is inherent in all membrane processes, since the permeation rates of different permeating components are different during membrane separation. For highly permeable selective membranes, the boundary layer effect is expected to be more significant. The decrease of concentration polarization means that the water concentration near the membrane/feed interface is close to the bulk water concentration. In this case, more water can be adsorbed on the membrane surface, thus improving the water flux. In addition, the decrease of concentration polarization reduces the transport resistance in the liquid boundary layer, which leads to the increase of water flux [39]. Qiu et al. [76] developed a mass transfer model based on the solution-diffusion theory with the consideration of concentration polarization to describe ethanol recovery by PV with PDMS membrane, as shown in Figure 11. Where C_b_ and C_bm_ are ethanol concentration in liquid bulk and the liquid boundary layer at the interface of liquid boundary layer/membrane, respectively. C_m1_ and C_m2_ are the concentration in membrane at interface of liquid boundary layer/membrane and on the downstream side of the membrane, respectively.

In air-swept PV, sweep velocity is known as a key parameter. For instance, increasing the scavenging speed can reduce the concentration and temperature polarization on the permeate side of the membrane, thereby resulting in a faster flow rate [77].

### 4.5. Simulation and Optimization of the PV Process

**Figure 12:**
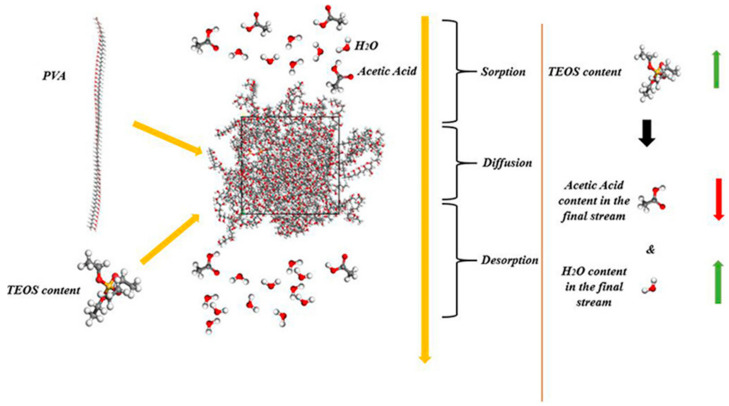
MDS of PV process of PVA/ tetraethyl orthosilicates (TEOS) membrane. Reproduced with permission from Reference [80]. Copyright 2018, Elsevier.

Molecular simulation technology is a newly developed computer-aided experimental technology that can be used to transform molecular-scale processes into visual processes. In recent years, molecular dynamics simulation (MDS) have often been employed for PV process evaluation, through the establishment of a reasonable model, and the separation mechanism of the membrane can be further understood. Kao et al. [78] systematically analyzed the influence of substituent structure on the free volume and PV performance of the aromatic PA membrane by positron annihilation spectroscopy (PAS) and MDS. The life and free volume size of the ortho-positronium obtained by the PAS test and MDS, and is consistent with the chemical structure of the PV membrane. Huang et al. [79] used MDS and positron annihilation lifetime spectroscopy to study the microstructure of various novel polyelectrolyte composite membranes (PECMS). These PECMS with different chemical structures were used in the PV dehydration of 90 wt % ethanol aqueous solution and it was found that their separation performance was related to their microstructure. Afterward, they analyzed the free volume shape and flexible stiffness of a polymer chain by MDS with radial distribution function and mean square displacement. Because the results are consistent with the simulation results, so it is proved that the separation performance of the PV membrane is related to the chemical structure of PECMS. Dashti et al. [80] used MDS and Monte Carlo simulation technology, combined with adaptive network-based fuzzy inference system and generic programming knowledge of artificial intelligence, and they studied the separation of water acetic acid by PVA silicon-based membrane under a wide range of experimental conditions (Figure 12). Using the method of molecular simulation, the results of all simulation models are compared.

In addition, many researchers use modeling methods to simulate the PV process. Recently, Rezakacemi et al. [81] carried out experimental and theoretical studies on the PV separation of cyclohexane (CX)/water by the PDMS membrane. Through experiments, they studied the effect of feed concentration on the performance of membrane PV, and the results show that the maximum separation coefficient of PDMS membrane and the feed concentration is 2500 and 80 wt % respectively. They also built a comprehensive mathematical model to predict the concentration of CX in the module, and the model was based on the coupling of the convection-diffusion method and Navier Stokes equation, whilst the finite element method was used to simulate the process. The results show that the model can better predict the concentration distribution and flow rate of CX in the process, and improve the separation efficiency by improving the convection mass transfer flux in the feed channel. In another study, Rezakazemi et al. [82] established a comprehensive mathematical model to evaluate the performance of PV, and the model was based on solving the conservation equation of water in the membrane module. The conservation equations including the continuity equation and momentum equation are derived and solved by the finite element method, then they used computational fluid dynamics software to solve the model equation and use the data to verify the model. The simulation results are in good agreement with the experimental data under different feed flow and feed temperature, which show that the permeation flux increases with the increase of feed flow and temperature.

For the optimization design of the PV process, Farshad et al. [83] purposed to find the optimal conditions to separate toluene from n-heptane mixture. A black box modeling using artificial neural network has been developed first, and then a multi-objective genetic algorithm has been employed to find the optimum condition based on the modeling results. The results showed that the experimental data are in good agreement with the predicted value of the model with the correlation coefficient greater than 0.99, and the mean square error of less than 1%. Both the model and the experimental data indicated that the increase of temperature and toluene concentration will increase the total flux and decrease the toluene selectivity respectively, while the increase of osmotic pressure will decrease the total flux and the toluene selectivity. Wee et al. [84] carried out an optimization study on the PV experiment of the IPA aqueous solution. It was found that when the temperature (75 °C), the feed concentration (94 wt %), the permeate pressure (1 KPa) and the feed flow rate (84 dm^3^/ h) were selected, optimal permeate flux (2.41 kg/m^2^h) and selectivity (1131) can be obtained. Given the separation of an azeotrope, low relative volatility mixture and point cut mixture, Naidu et al. [85] explored the structure and parameter optimization of the continuous mixed distillation PV process with different membrane components including series, parallel and series-parallel. For the separation of the IPA water mixture (azeotropic separation), the series structure of membrane module was found to be more economical than the parallel or series-parallel structure. While propylene propane separation (closed boiling) and acetone water separation (tangential pinch), the parallel structure of the membrane module was considered more economical. Cojocaru et al. [86] used an optimization method combining the factor modeling and analysis with expectation function in the multi-response optimization of the PV process. The total factor design of water/acetonitrile and water/ethanol mixed solvent was carried out, and the main influence and interaction of each factor were determined through factor modeling and analysis. In addition, overlapping graph, factor model and function method are used to determine the appropriate range and optimal operation conditions of PV process.

## 5. Applications of Polymeric Membranes for PV Separation

With the progress of science and technology and the development of industrialization, PV membrane separation technology has been more and more widely used. This section mainly introduces applications of polymeric membranes for PV separation in chemical, desalination, petrochemical, medicine, food and biotechnology industry.

### 5.1. Chemical Separation

PV process has widely been used in chemical separation, which mainly includes dehydration of organics, recovery of organics from water and separation of organic mixtures.

The main industrial application of PV is the dehydration of organic liquid. By modifying the active layers of PV membranes with different chemical components and structures, these membranes exhibited much improved flux and selectivity toward water extraction [64,87]. Chung and his colleagues pioneered the development of polybenzimidazoles (PBI) based on PV membranes [88,89]. They have prepared flat and hollow fiber PBI membranes for dehydration of various solvents, such as alcohol, glycol, and acetone [62].

In addition, esterification as a reversible reaction to produce organics and water, in order to improve the yield of esterification reaction, it is necessary to remove water from the product. PV membrane reactor is a kind of selective membrane, which is used to remove water from the mixture of esterification reaction and obtain a high yield of ester. Korkmaz et al. [90] discussed the esterification of acetic acid with isobutyl alcohol under different types of membranes, in which PDMS and PVA membranes with permeability achieved the best experimental results. In another example, Zhang et al. [91] improved the conversion of esterification of acetic acid and n-butanol by catalytic active PV membrane. Recently, esterification has been further strengthened, and the PV of lipase-catalyzed chemical esterification to assist the production of enzyme esters has attracted widespread attention. For example, Krishna et al. [92] studied the esterification of isoamyl alcohol with acetic acid in n-heptane solvent catalyzed by immobilized Mucor oryzae lipase. They observed that the conversion could reach higher than 95% even in very low enzyme concentrations. Ziobrowski et al. [93] introduced the production of glycerin monostearate by enzymatic method in different high polar organic solvents to remove the water produced in the process of PV esterification. Koszorz et al. [94] investigated the kinetics of enzymatic esterification of oleic acid, isoamyl alcohol, and the main compounds of fusel oil.

**Figure 13:**
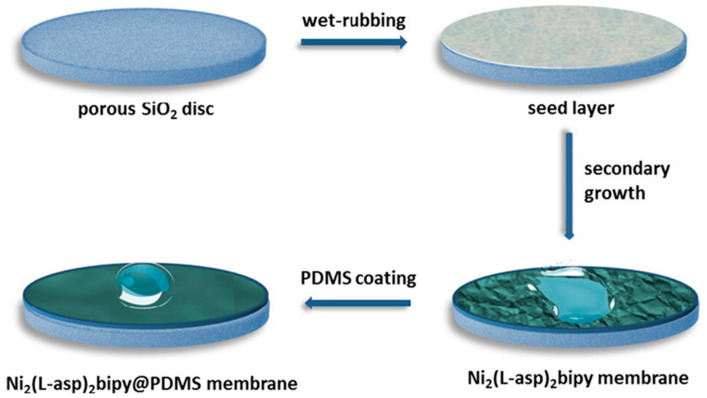
Schematic illustration of the preparation process of Ni_2_(L-asp)_2_bipy and Ni_2_(L-asp)_2_bipy@PDMS membranes and water/ethanol separation on them. Reproduced with permission from Reference [97]. Copyright 2017, Elsevier.

Another major application of PV is the recovery/removal of organics from water. PV has been verified to be used to recover aromatic compounds from dilute aqueous solution and remove volatile organic compounds from wastewater. As a volatile substance, ethanol recovery from wastewater is very common in the application, for example, Haris et al. [95] successfully prepared the laterite zeolite-geopolymer PV membrane for the separation of ethanol-water but the purity of ethanol is affected by the selectivity of the membrane. However, experimental results showed that the hydrophobic materials coated on the zeolite-based polymer membrane can effectively reduce the hydrophilicity of the polymer, thereby the PV system becomes more feasible for ethanol purification. Sunitha et al. [96] prepared a compact chitosan membrane and crosslinking it with phosphoric acid at different time intervals to separate ethanol-water. They studied the PV performance of the as-prepared membrane under different operating conditions. The results indicated that the crosslinking time, the feed composition, the membrane thickness and the osmotic pressure affect the permeability flux and selectivity of the membrane, respectively. With the increase of influent concentration, the performance of the membrane was significantly affected by the increase of the swelling degree of polymer, which leads to an enhancement of the flux and a decrement of the selectivity. The increase of osmotic pressure leads to the decrease of membrane flux and selectivity, while the increase of membrane thickness reduces the increase of flux and selectivity. At 95.58 wt % ethanol concentration, the phosphorylated chitosan has the potential to break the azeotropic barrier of ethanol, which is very stable in the aqueous solution below 4% and the phosphorylation can improve the selectivity without large flux loss. In the application of recovery of organics, ethanol with final purity higher than 99.5% can be obtained by PV. It is worth mentioning that Wang et al. [97] in order to evaluate the effect of metal-organic framework surface wettability for the purification of ethanol from water/ethanol mixtures. The hydrophilic Ni_2_(L-asp)_2_bipy membrane is switched to hydrophobic Ni_2_(L-asp)_2_bipy@PDMS membrane via vapor deposition of PDMS. The stable Ni_2_(L-asp)_2_bipy membrane exhibits a high flux of water and an acceptable separation factor. The polycrystalline Ni_2_(L-asp)_2_bipy membranes were fabricated on porous SiO_2_ discs by a seeding-secondary growth method as shown in Figure 13. In addition, Solak et al. [98] studied the PV separation of acetone and water by sodium alginate (NaAlg)/polyvinylpyrrolidone (PVP) membrane in the concentration range of 0–100 wt %. The membranes were prepared by crosslinking CaCl_2_ with different ratios of NaAlg to PVP and the effects of operating temperature, feed composition and membrane thickness on PV performance were studied. It was found that the permeation rate increased with the increase of PVP content, but the separation factor did not change significantly.

PV technology has also been proved to replace distillation and extraction to separate benzene (BZ) and CX solution whose volatility difference is only 0.6 °C, so as to reduce the operation cost. The adsorption selectivity was found to be determined by the affinity between the double bond of BZ and the polar group of polymer membrane [99], and polymers with polar groups can promote BZ permeation through the membrane. For example, Yamasaki et al. [100] suggested that the utilization of membranes with hydrophilic groups could improve the selectivity of BZ. In addition, Han et al. [101] prepared a membrane for PV separation of methanol (METH) / MTBE using polyarylsulfone and cardo as membrane materials, and the swelling and mechanical properties of the membrane in METH/MTBE mixture were studied. They found that the permeation flux increases or METH selectivity decreases with the increase of feed temperature.

### 5.2. Desalination

RO desalination is an important step of wastewater reuse as it can remove salts and trace contaminants. However, RO usually generates high salinity brines that need to be dealt with. MD, a process largely unaffected by salinity, provides a way to treat desalination brines to high water recovery and has been proposed as a solution for RO brine management. However, pore wetting of membranes in MD is one of the major hurdles that prevents its implementation in wastewater treatment systems, as amphiphilic organic compounds present in wastewater can lead to pore wetting and loss of selectivity over time [102]. Compared with traditional desalination processes, PV membrane separation technology has a higher desalination rate. This sub-section mainly reviews the inorganic membrane, organic membrane and hybrid membrane in the application of seawater desalination.

Inorganic membranes are advantageous for high separation performance, good thermal, chemical stability and low fouling tendencies [103]. In recent years, researching on inorganic membranes such as zeolites and amorphous microporous silica has become more and more active. Based on molecular size and shape, the rigid ceramic structure and precise tuning of the pore sizes endow inorganic membranes great capabilities for separation processes. For example, Singh et al. [104] proposed cetyltrimethylammonium bromide (CTAB)—silica membrane with flat and hollow fiber polysulfone structure. At 25 °C, when 40 g/L NaCl seawater is pervaporated by the membrane and the salt resistance rate can reach 99.9%. However, when the temperature exceeds 40 °C, the permeate flux increased greatly by an order of magnitude while with much less salt-rejection efficiency. This could be attributed to the disturbance in the barrier layer of the mesostructure under the influence of higher temperature, that solvated NaCl molecules might have slipped in the permeate side since the barrier layer formed by interaction of CTAB surfactant with silica is through a weak electrostatic interaction. Upon cooling back to room temperature, the salt-rejection efficiency (99.9%) of the membrane was restored. Furthermore, various organic membranes have been widely used in seawater desalination. For example, Korngold and his colleagues [46] prepared a hydrophilic polyethylene-based ion-exchange hollow fiber membrane and it was applied to the study of increasing the air humidity of the PV desalination process. It was found that the diffusion resistance of water through the membrane was the main limiting factor, but increasing the charge density and reducing the membrane thickness, the water flux can be improved. Zwijnenberg et al. [52] adopted a new membrane process, in which solar-driven PV was used for the production of distilled water from untreated sea-water and formation water. The configuration used consisted of direct solar single effect membrane PV unit and dense tubular membranes, which were made of 40μm thick modified PEA (polyether amide-based polymer) film. In all cases retention of typical ions as sodium, chloride, calcium as well as specific problematic ions (arsenic, boron and fluoride) was higher than data reported for pressure driven membrane processes such as NF and RO. The system was designed to minimize capital cost and the high cost of pre-treatment fouling feed streams. In another case, the DuPont company developed a homogeneous and dense hydrophilic polyether ester membrane with excellent chemical resistance and strong mechanical strength. The membrane can effectively intercept borate, selenate and sodium chloride, and the recovered polluted water and low concentration saltwater, so it can be used in agricultural production [39]. The desalination capabilities of a hydrated cellulose membrane and a bacterial cellulose membrane prepared from plant cellulose (wood or cotton) were discussed by Naim et al. [105]. Strong hydrophilic CA membrane was prepared by the basic phase transformation theory, although the membrane has asymmetric morphology, it has high desalination rate and large flux.

**Figure 14:**
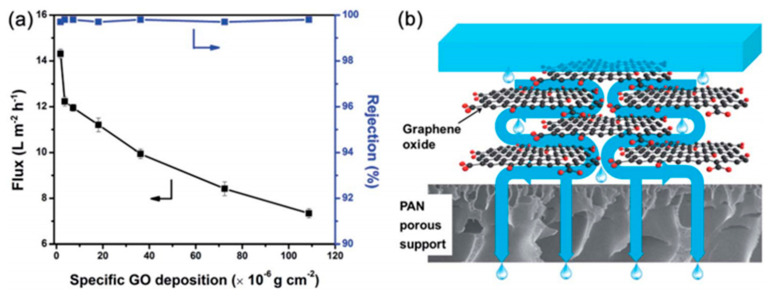
(**a**) Effects of specific graphene oxide (GO) deposition amount on the PV performance of GO/PAN membranes and (**b**) schematic representation of the mechanism for water molecule transport through GO sheets. Reproduced with permission from Reference [75]. Copyright 2015, the Royal Society of Chemistry.

Different kinds of composite membranes have also been produced and utilized for the PV desalination process. For instance, Liang et al. [75] adopted a simple vacuum filtration-assisted assembly method to deposit graphene oxide (GO) films with two-dimensional nanochannels on a functionalized PAN UF membrane to prepare a PV composite membrane. These GO/PAN composite membranes presented great potential for desalination applications by PV. Under their testing conditions, these PV composite membranes exhibited a high water flux up to 65.1 L m^−2^ h^−1^ with a high rejection for desalination (about 99.8% at 90 ℃). It is noteworthy that the composite membranes retain their high performance when treating high salinity water with salt concentrations up to 10^5^ ppm. In the process of experiment, they found a significant decrease in the water flux was observed with increasing GO loading above 5 μg cm^−2^ (Figure 14a), which could be attributed to the higher mass transfer resistance as the transport path of water molecules increased with the thicker deposition of the GO films. In general, as the mass transfer resistance increases, the membrane efficiency drops [106]. However, the rejection of the GO/PAN composite membrane was still maintained at over 99.7% with increasing GO layer thickness. Therefore, minimizing the thickness of the GO layer while maintaining its structural integrity is crucial in fabricating high-efficiency PV membranes. As shown in Figure 14b, the “ideal” pathway for water molecule transport through the tortuous nanocapillaries between the well stacked GO sheets [107,108,109], where the GO inter-sheet spacing determines the selectivity performance of the membrane.

Ling et al. [74] employed electrospray/electrospinning technology to deposit three layers of nanofiber PV composite membrane. The as-synthesized membrane effectively reduced the mass transfer resistance and increased the porosity allowing a stable separation efficiency. Xie et al. [73] synthesized a cross-linked PVA hybrid membrane containing highly dispersed inorganic silica through the sol-gel method, which improved the desalination and separation performance of PVA hybrid membrane as well as its stability in water.

### 5.3. Petroleum Chemical Industry

With the acceleration of urbanization, industrial wastewater has become one of the main sources of urban pollution. There are not only volatile organic compounds, but also various salts in industrial wastewater. In the field of petrochemical industry, the salt solution and organics in wastewater can be separated by the use of PV membrane separation technology. For example, the recovery of p-xylene from xylene isomers is a significant step in large-scale petrochemical synthesis, because p-xylene is an important chemical raw material for the synthesis of terephthalic acids and their downstream productions (polyester resin and fiber) [110,111]. Besides, PV membrane separation technology can be used to separate the organic mixtures, in which different contents are existed in the form of an azeotrope, near azeotrope as well as isomer, and those are very difficult to be isolated via general distillation.

**Figure 15:**
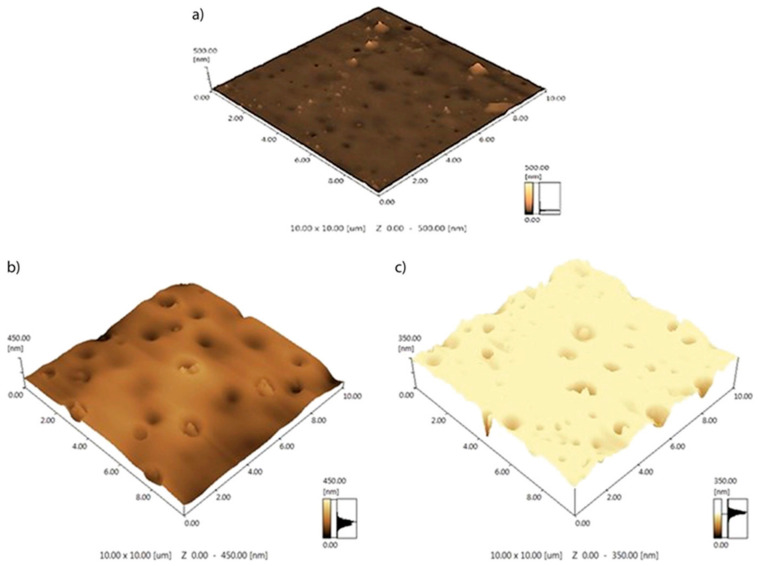
AFM images of (**a**) the pure PVB membrane, (**b**) the PVB membrane with 2 wt % SiO_2_, and (**c**) the PVB membrane with 2.5 wt % SiO_2_. Reproduced with permission from Reference [112]. Copyright 2019, the Wiley Online Library.

PV as a new technology for gasoline desulfurization. More and more attention has been paid to the method of removing eco-friendly sulfur in the petrochemical industry because of high selectivity, feasibility, economy and safety of PV membranes. Commercial gasoline is a complex mixture of alkanes, C_5_-C_14_ alkenes, cycloalkanes and aromatics, and it consists of products from isomerization, reforming and fluid catalytic cracking (FCC) units. FCC gasoline not only accounts for 30–40% of the total gasoline, but also it contains 85–95% sulfur element. Therefore, desulfurization from FCC is a key to achieve deep desulfurization of gasoline. Recently, PV has exhibited several potential advantages in FCC gasoline desulfurization. For example, Hou et al. [112] improved the performance of the PV membrane by adding SiO_2_ nanoparticles into polyvinyl butyral (PVB), and the coupling agent was added to enhance the compatibility between SiO_2_ and PVB to avoid membrane defects. They also tested the actual FCC gasoline by studying the effects of SiO_2_ content, operating temperature and active layer thickness of PVB/SiO_2_ performance. Figure 15 shows the three-dimensional AFM images of the pure PVB and PVB/SiO_2_ composite membranes. The roughness of the surface membranes, which is the distance of the two tiny peaks or valleys on the surface of the membrane, was measured. The average roughness Ra (arithmetical mean deviation of the assessed profile) of the pure PVB membrane is 5.2 nm, and it increases with increasing SiO_2_ content. The average roughness values of the PVB membrane with 2 and 2.5 wt % SiO_2_ are 24.1 and 34.1 nm, respectively. A rough surface increases the contact area between the membrane and the gasoline, which is beneficial to an increase of the flux. On the other hand, as shown in Figure 15c, an excessive SiO_2_ content leads to increased defects in the membrane. There are many irregular depressions on the surface of the membrane which can reduce the selectivity of the membrane. The diffusion rate of gasoline components in the membrane increases with the higher temperature, which leads to the increase of permeation flux. Considering the concentration factor and the working temperature of flux, 80 °C is selected as the best working temperature. In conclusion, when the mass ratio of SiO_2_ to PVB is 2wt %, the sulfur enrichment coefficient reaches 3.94 and the flux is 1.44 kgm^−2^ h^−1^ at 80 °C. This research provided useful information on the further development of PV membrane technology for the separation of sulfur compounds.

### 5.4. Medicine Separation

PV technology is closely related to medicine and chemical industry. In the process of drug production, organic solvents such as ethanol, IPA, butanol, acetone, and butyl acetate are widely used in extraction, cleaning and reaction solvents. It is necessary to meet the requirements of solvent recycling in the use of these organic solvents. PV as a new type of environmental protection and energy-saving separation technology, which has a wide range of applications and prospects in the field of medicine and chemical industry.

**Figure 16:**
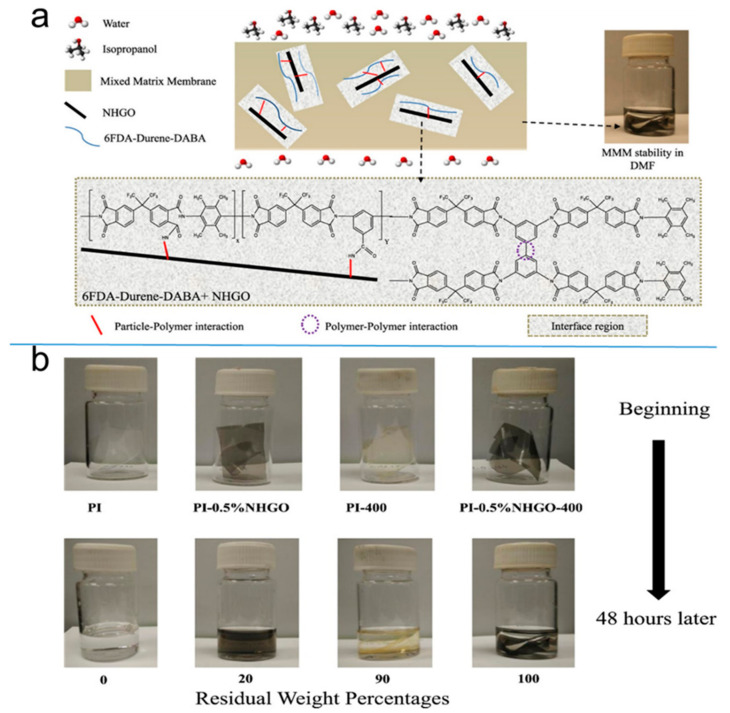
(**a**) Possible chemical reactions between NHGO particles and 6FDA–Durene-DABA and possible chemical evolution during thermal treatment at 400 °C. (**b**) Dissolution results of PI and various mixed matrix membranes (MMMs) in DMF after 48 h. Reproduced with permission from Reference [67]. Copyright 2017, the Elsevier.

IPA is widely used as a solvent in the synthesis of cephalosporins and other drugs. However, due to the complexity of solvent components and the co-boiling point of IPA and water at 12.6% water mass fraction, the recovery of IPA becomes very difficult. Due to high energy consumption and waste generation, traditional methods for the separation of IPA/water mixture including azeotropic distillation and extractive distillation are restrained. Recently, a novel MMM consisting of a cross-linkable 6FDA PI matrix and ammonia functionalized GO (NHGO) particles has been molecularly designed at elevated temperatures for water/IPA separation by Salehian et al. [67]. Possible chemical reactions between NHGO particles and 6FDA–Durene-DABA at 400 °C were proposed, as shown in Figure 16a. The pristine PI dissolves in dimethylformamide (DMF) completely within a few hours, whereas some residues stay in the bottle of PI-0.5%NHGO (Figure 16b). Surprisingly, the thermally treated samples remain as films even after two days. The results show that a promising future of PI-0.5%NHGO-400 composite membranes for the dehydration of IPA via the PV process. In another case, Slater et al. [113] evaluated the effect of PV as a green drying process to recycle solvent tetrahydrofuran in drug synthesis, and this study has been applied in the synthesis of a new tumor drug step.

### 5.5. The Food Industry

**Figure 17:**
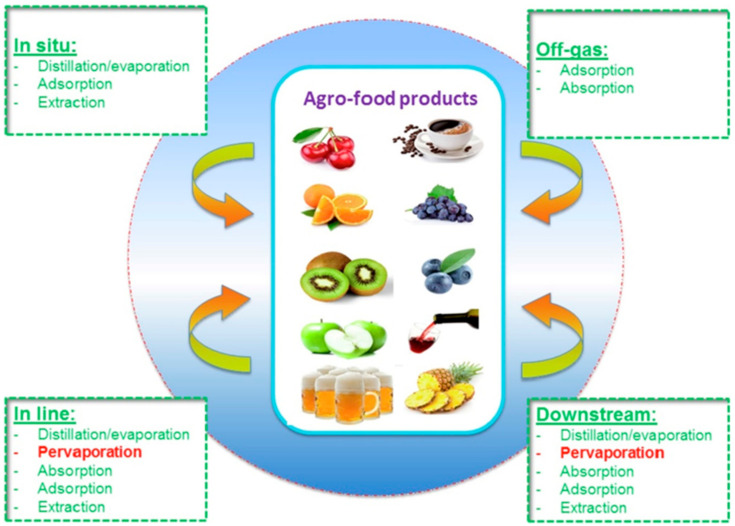
Techniques used for the aroma recovery from food products. Reproduced with permission from Reference [56]. Copyright 2019, the Elsevier.

PV membrane separation technology has a broad application prospect in food processing. Potential application examples in food applications include recovery and concentration of aromatic compounds, the dealcoholization of alcoholic beverages and dehydration of azeotropic mixtures. To date, several recovery techniques have been proposed for such a task, as indicated in Figure 17.

Nowadays, the production of nonalcoholic or low alcohol beverages with alcoholic beverages is a great challenge for food technicians, because drinking a lot of alcoholic beverages may bring health problems to consumers. Although some sensory substances will be lost in the process of dealcoholization, it still makes the dealcoholization process highly sensitive to maintain the quality characteristics of the beverage. Therefore, membrane-based separation technology using a permeable selective barrier has been highly promoted. As a multi-component colloidal solution, alcoholic beverages (such as beer and wine) complicate the removal of alcohol. PV is likely to be achieved in any dealcoholization process, as it can restore aroma and meet the quality requirements of non-alcoholic beverages. In addition, hydrophobic membranes are required in PV technology if ethanol removal is efficient [114].

For example, Aroujalian and Raisi [115] extracted the volatile aroma components of EtAc, ethyl butyrate, hexanal, limonene, linalool and α-terpineol from orange juice through the PV process. Especially, the PDMS membrane has a better enrichment effect on the extraction of ethyl butyrate and hexanal when the real space is increased. In the food industry, unit operations of stabilization (such as blanching) produce aqueous effluents generally non-polluting but often odorous. Therefore, Souchon et al. [116] applied the PV process to the deodorization of a cauliflower blanching effluent to reduce its volatile organic compounds content and to try to recover a valuable food flavoring fraction. A systematic study of PV has been performed on three (S-methyl thio-butyrate, dimethyl trisulfide and dimethyl disulfide) sulfur compounds identified as typical compounds of the cauliflower odor, then, the separation performances were evaluated on an industrial effluent through physicochemical and sensorial analysis. They showed that PV was an efficient process for deodorization and offers a real potential for the valorization of the permeate. In another case, Figoli et al. [117] proved that the PDMS membrane can recover linalool and linalool from bergamot oil, which can provide characteristic essence for bergamot fruits

### 5.6. Biotechnology

**Figure 18:**
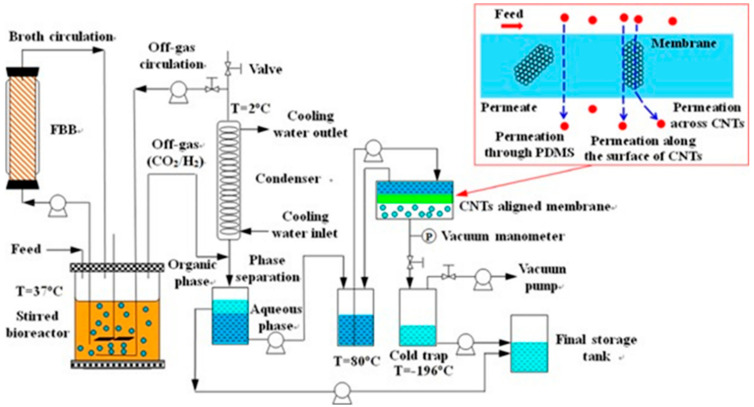
Schematic diagram for acetone, butanol and ethanol (ABE) fermentation coupled with the hybrid gas stripping-PV process. The inset shows butanol permeation assisted by CNTs through the membrane. Reproduced with permission from Reference [118]. Copyright 2016, the Wiley Online Library.

PV technology has potential application prospects in the recovery of biobutanol from biomass acetone, butanol and ethanol (ABE) fermentation broth, which has attracted extensive attention. For example, a novel two-stage gas stripping PV process integrated with ABE fermentation was developed for butanol recovery by Xue et al. [118], with gas stripping as the first stage and PV as the second stage using the carbon nanotubes (CNTs) filled PDMS MMMs. The schematic diagram for ABE fermentation coupled with the hybrid gas stripping-PV process is shown in Figure 18. This gas stripping PV process with less contaminated risk is effective in increasing butanol production and reducing energy consumption.

Moreover, Liu et al. [119] used PDMS/ceramic composite PV membrane to recover butanol from aqueous solution. They studied the effects of operating temperature, feed concentration, feed flow rate and operating time on membrane PV performance, and it was found that with the increase of feed temperature and butanol concentration, the total flux of the membrane increased and the separation factor decreased slightly. With the increase of feed flow, the total flow increases gradually, and the separation coefficient changes little, and the as-obtained PV membrane has high flux, so it is suitable for the recovery of butanol from ABE fermentation broth.

## 6. Conclusions and Outlooks

In summary, we demonstrated recent development of the synthesis and functionalization of polymeric membranes for PV separation applications. It is clear that PV separation technique revealed several advantages including simplicity, flexibility, low cost, as well as high energy and separation efficiency for membrane-based separation applications. In addition, the functionalization of polymeric membranes with other organic materials, nanoparticles, and carbon nanomaterials extended the separation applications and improved the performance of hybrid polymeric membranes. This work will be helpful for readers to understand the principles and applications of PV-based chemical and biological separations based on functionalized polymeric membranes.

Although great development on the fabrication of various polymeric PV membranes for separations has been done in the last years, there are still some spaces that should be filled in the future. Firstly, more efforts should be directed to the chemical modification of polymeric materials as well as the incorporation of novel fillers or the reinforcement of existing ones through a chemical modification to improve the specific components transport and the selectivity of PV membranes toward specific separation applications.

Second, the membrane fouling issue due to the deposition/adsorption of suspended particles, colloids and organic/inorganic matters is one of the key obstacles limiting the broader applications of PV. Therefore, the preparation of polymer membranes with low-fouling potential has been attracting great interest. To achieve this aim, it is possible to form polymer membranes with hierarchical surface nanostructure through molecular printing and nano-lithography techniques. In addition, the improvement of the anti-fouling performance of polymer membranes could be improved by modifying the membrane with hydrophobic nanomaterials.

Third, for the seawater desalination with PV process, low-grade heat energy or renewable energy such as solar energy to heat the feed liquid can be a promising alternative to conventional means where energy was obtained from fossil fuels and thus desalination inevitably involve greenhouse gas emissions. Therefore, the exploration of renewable resources and the utilization of low-grade heat energy instead of fossil fuels will be new development directions toward energy-efficient and eco-friendly PV desalination in the future.

Fourth, the integration of PV with systems for reaction or separation should be further developed based on the potential of available membranes for separation, which can substantially improve the reaction efficiency, yield and process economy. In this case, the performance of the utilized membrane materials is crucial for the integration of PV membrane systems, and therefore more efforts should be performed to study the effects of material structure, properties, and functions on the separation efficiency of PV membranes.

In addition, novel polymeric membranes could be fabricated by combining polymer matrix with other functional nanomaterials such as solar-sensitive nanoparticles and 2D materials, which will extend the applications of the fabricated membranes in various separation techniques beyond PV. For example, it will be very interesting to conjugate polymers with TiO_2_ and ZnO nanoparticles to improve the photo-degradation ability of the polymer membranes to extend their applications in environmental science. Meanwhile, the combination of polymers with graphene and MXene materials for creating novel hybrid membranes could be carried out.

## Figures and Tables

**Table 2:** The different operation mode of PV is summarized from the characteristics and range of application.

Operation Mode of PV	Characteristics	Range of Application	References
Vacuum PV	• High efficiency but high energy consumption • The vacuum pump has a large load • Valuable permeates cannot be recovered	• Removal of hazardous volatile organic compounds from water • Production of acetone, butanol and ethanol (ABE) from lignocellulose • Dehydration of ethyl acetate (EtAc)	[63,64,65]
Thermal PV	• This method is often used in combination with vacuum PV in industry. The mass transfer force and cost are smaller than the vacuum PV, but the separation efficiency is low. • This method cannot guarantee to remove the non-condensable gas from the system, so it is rarely used in actual production.	• Ethanol dehydration • IPA dehydration	[66,67,68]
Carrier gas purging PV	• The carrier gas is recycled.	• Continuous separation of ternary mixtures by microfluidics • Recovery of organic components from wastewater	[69,70]
Condensable carrier gas purging PV	• Low-pressure water vapor can be used as a purge gas. • After condensation, water separated from the permeating component and water was evaporated for recycling.	• removing low concentration organic solvents from water	[71]
